# The Effect of Obesity on the Waist Circumference Cut-Point Used for the Diagnosis of the Metabolic Syndrome in African Women: Results from the SWEET Study

**DOI:** 10.3390/ijerph191610250

**Published:** 2022-08-18

**Authors:** Philippe J. Gradidge, Shane A. Norris, Nigel J. Crowther

**Affiliations:** 1 Centre for Exercise Science and Sports Medicine, Faculty of Health Sciences, University of the Witwatersrand, Johannesburg 2050, South Africa; 2 SAMRC/Wits Developmental Pathways for Health Research Unit (DPHRU), Faculty of Health Sciences, University of the Witwatersrand, Johannesburg 2050, South Africa; 3 Department of Chemical Pathology, National Health Laboratory Service, Faculty of Health Sciences, University of the Witwatersrand, Johannesburg 2050, South Africa

**Keywords:** metabolic syndrome, obesity, waist circumference, sub-Saharan Africa

## Abstract

Waist circumference (WC) is one of the diagnostic criteria for metabolic syndrome (MetS). However, studies have shown that the waist cut-point may be influenced by BMI. The aim of this study was to, therefore, determine whether the presence of obesity influences the WC cut-point used to diagnose MetS in sub-Saharan African women. The second aim was to determine whether calculated cut-points of other waist-related and dual-energy X-ray absorptiometry (DXA)-determined anthropometric measures used for the diagnosis of MetS were also influenced by BMI. Biochemical, simple anthropometric and dual-energy X-ray absorptiometry-derived anthropometric data were collected in 702 black South African women from the Study of Women Entering and in Endocrine Transition (SWEET). A receiver operating characteristic curve analysis was used to determine waist, waist-to-hip (WHR) and waist-to-height ratios, body shape index (ABSI), total body fat, trunk fat, and peripheral (arm + leg) fat cut-points for MetS (without waist) in subjects with BMI above or below the median value. The estimated WC cut-points (107 cm, 93.5 cm) for women with high BMI and low BMI, respectively, and the cut-points for the other anthropometric variables for the diagnosis of MetS were greater in high BMI women compared to low BMI women. The exceptions were WHR and ABSI, for which the cut-points were very similar in both BMI groups, and peripheral fat, where the cut-point was lower in the high BMI group. Logistic regression analysis demonstrated that WC was associated with a higher risk (odds ratio [95% CIs]: 1.07 [1.04, 1.10]; *p* < 0.0001), whilst hip was associated with a lower risk (0.97 [0.94, 0.99]; *p* = 0.02) for MetS. These data suggest that with increasing BMI, the higher levels of protective gluteofemoral fat lead to the requirement for higher WC cut-points for MetS diagnosis. The opposing associations of waist and hip with MetS risk make WHR a more appropriate variable for diagnosing MetS among African women as the WHR cut-point is less influenced by increasing BMI than is WC, which was also observed for ABSI.

## 1. Introduction

The metabolic syndrome (MetS) is a phenomenon characterized by a clustering of various cardiometabolic diseases, such that individuals with at least three of the five components are at an increased risk of cardiovascular disease (CVD) and associated early mortality. A comprehensive review of MetS demonstrated that women living in sub-Saharan Africa (SSA) are particularly vulnerable to the syndrome compared with other countries, mostly as a direct consequence of the increasing obesity pandemic in the region [1]. Recent pooled data from fourteen different SSA studies show that the prevalence of MetS ranges from 11% to 24% [2]. African populations in the southern region of SSA have the highest prevalence of MetS (28.7% (95%CIs: 18.5%, 40.0%) compared with central (7.2% (0.6%, 20.1%), eastern (10.0% (4.7%, 17.3%), and western (18.0% (12.7%, 24.0%) SSA [2]. This study also demonstrates that African females have a higher prevalence of MetS compared with African males (21% versus 9%, respectively) and show that rural dwelling populations are less vulnerable to MetS than urban populations (11% versus 16%, respectively) [2]. The relatively high prevalence of MetS in southern SSA is consistent with the higher presence of obesity in this African region compared with other SSA countries [3]. Ekoru et al. confirmed that the syndrome is mostly driven by central fat in African women and stressed the need for an effective detection of CVD risks using accessible means [4].

Data from studies conducted in various African regions demonstrate that some components of MetS are more prominent in African populations [5,6,7]. These studies showed that high waist circumference and low high density lipoprotein cholesterol (HDL) levels are the most prevalent components, which was further confirmed in a recent study of MetS in black South African women [8].

Waist circumference (WC) is a simple and inexpensive indicator of central obesity and a much stronger determinant of CVD risks compared with the body mass index (BMI) [9]. Waist circumference is one of the diagnostic components of MetS; however, it has strong independent associations with each of the individual cardiometabolic factors that constitute MetS [10] and it is becoming increasingly evident that the recommended WC cut-points, 80 cm and 94 cm, derived from European populations and used for detecting MetS in females and males, respectively, are unsuitable for use in SSA [5,11]. Furthermore, the WC cut-point is influenced by the presence of obesity as noted in the meta-analysis conducted by Ekoru et al. [4] in SSA populations. Therefore, the first aim of this study was to determine, using data from a single population, whether the WC cut-point used for diagnosing MetS changes with increasing BMI. The second aim was to determine whether calculated cut-points of other waist-related and dual-energy X-ray absorptiometry (DXA)-determined anthropometric measures used for the diagnosis of MetS are also influenced by BMI. This study was performed in a cohort of African women with a known high prevalence of obesity and associated cardiometabolic disease risk factors [8].

## 2. Materials and Methods

### 2.1. Study Participants

This cross-sectional study included women from the Birth to Twenty Plus (BT20) cohort. The BT20 study is the longest-running longitudinal study of health determinants in Africans, which began in 1990, with a sample of 3273 participants. The women used in the current study were mothers or care givers of the index children who were recruited from Soweto, Johannesburg into the longitudinal study in 1990. Both mothers, care givers, and children have been followed-up at regular intervals from the beginning of the study, from whom 867 of the former were eligible for recruitment into this study based on age (40–60 years old), not pregnant, and black African female. A number of these women refused to participate (*n* = 79), died (*n* = 37), were terminally sick (*n* = 3), or were untraceable (*n* = 46). The final sample included 702 women that provided written informed consent. Ethical approval was given by the Human Research and Ethics Committee (Medical), University of the Witwatersrand (M110627). This sub-group of 702 women constitutes the Study of Women Entering Endocrine Transition (SWEET) [8].

### 2.2. Body Composition

Anthropometric measurements taken in this study have been described before, but they included simple anthropometry, i.e., BMI, waist circumference (WC), hip circumference (HC), waist-to-hip (WHR) and waist-to-height ratios (WHtR), and DXA-derived body composition measures, i.e., sub-total fat, trunk fat and arm and leg fat [8]. A Body Shape Index (ABSI) was calculated using the following formula: ABSI = WC/BMI^2/3^ × height^1/2^ [12].

### 2.3. Biochemical Analysis

The methods used for measuring the fasting glucose, high density lipoprotein cholesterol (HDL) and triglyceride levels, and systolic and diastolic blood pressure have been described in detail previously [8]. The presence of MetS and its five components were defined using the harmonised guidelines [13]. The MetS was also diagnosed without WC by the presence of 3 of the 4 remaining criteria as follows: fasting blood glucose ≥ 5.6 mmol/L; fasting triglycerides ≥ 1.7 mmol/L; HDL < 1.3 mmol/L, and elevated blood pressure (systolic BP ≥ 130 mmHg or diastolic BP ≥ 85 mmHg) [13].

### 2.4. Statistical Analysis

Statistical analyses were performed using Stata/SE for Windows, version 17.0 (StataCorp, College Station, TX, USA). The normally distributed continuous data are presented as a mean ± SD in tables, otherwise a median (interquartile range) was used for skewed continuous data. The categorical data are presented as a mean (95% CIs). A Student’s unpaired t-test for continuous data or an χ^2^ test for categorical data were used to compare the variables between BMI groups. A receiver operating characteristic (ROC) curve analysis was used in conjunction with the Youden index to determine the optimal cut-points for the WC, WHR, WHtR, BMI, subtotal fat, arm and leg fat, and trunk fat for the diagnosis of MetS. These analyses were conducted in women above and below the median BMI (32.8 kg/m^2^). The median BMI was used to generate these 2 groups to ensure the maximal sample size for each group and to rule out the possibility that any differences observed between the 2 groups in the outputs from the ROC curve analyses were driven by differences in the sample sizes. The sensitivity, specificity, and area-under-the-curve were also calculated from the ROC curve analyses for each of the anthropometric variables. A logistic regression analysis with an adjustment for age was used to determine the association of waist and hip with the MetS risk.

## 3. Results

### 3.1. Descriptive Characteristics

The data in Table 1 shows that when the study participants were divided into two groups based on the median value for BMI, all anthropometric variables, as would be expected, were significantly higher (*p* < 0.0005 for all except WHR, *p* < 0.005) in the subjects in the upper half. The systolic (*p* < 0.005) and diastolic (*p* < 0.0005) blood pressure were higher in the upper half, whilst the HDL was lower (*p* < 0.0005). The fasting blood glucose and triglyceride levels were not significantly different between the groups, nor was the prevalence of high fasting glucose and high triglyceride levels. The prevalence of elevated blood pressure, WC, and low HDL were significantly higher (*p* < 0.0005 for all) in women who had a BMI above the median value (Table 1). When MetS was diagnosed using either the standard harmonised guidelines, i.e., the presence of three or more of the five criteria [8], or the modified guidelines requiring three or more out of the four criteria (excluding WC), women with BMI above the median value had a significantly higher prevalence of both forms of the MetS (*p* < 0.0005 for both).

### 3.2. The Effect of BMI on WC Cut-Points for MetS

When the study population was divided into two groups based on the median BMI value and the ROC curve analysis was used to determine the optimal cut-points of the different simple anthropometric variables for the diagnosis of MetS, the WHR and ABSI were the only variables that gave similar cut-points in those with a lower (WHR, 0.85; ABSI, 0.127) or higher (WHR, 0.84; ABSI, 0.129) BMI (Table 2). All other variables, i.e., BMI (35.9, 29.3 kg/m^2^), WC (107, 93.5 cm), and WHtR (0.66, 0.58), gave much higher cut-points in the higher compared to the lower BMI group, respectively. With regards to the AUC for the ROC curve, in the lower BMI group, the WHR had the third highest AUC (0.72) with the highest WC and ABSI (both 0.74), whilst in the higher BMI group, the WHR had the highest AUC of 0.62 compared to ABSI (0.59), which was second, and a WC (0.58), which was third (Table 2). In addition, the WHR had the second highest sensitivity for MetS diagnosis (0.70) after ABSI (0.86) and the same specificity as the WC and WHtR (0.52) when used in the lower BMI group, with ABSI having the lowest specificity at 0.38. In the higher BMI group, the WHR had the third highest sensitivity (0.59) with ABSI having the highest (0.66) with BMI second (0.60) and the second highest specificity (0.52) with the WC being slightly higher at 0.53 and ABSI at 0.45.

When using the DXA-derived anthropometric measures, both the subtotal (39.5, 25.9 kg) and trunk fat (13.8, 12.1 kg) had higher cut-points in the higher compared to the lower BMI group, respectively, whilst this trend was reversed for the arm and leg fat (11.0, 15.1 kg) (Table 3). In the group with the lower BMI, the trunk fat had the highest AUC (0.70) and the highest sensitivity (0.68) and specificity (0.52). In the higher BMI group, the subtotal fat had the highest AUC (0.57) and the highest sensitivity (0.56) and specificity (0.51).

### 3.3. The Relationship between Waist and Hip Circumference and Risk of MetS

The data from the ROC curve analysis (Table 2 and Table 3) show that the WHR gave similar cut-points for the diagnosis of MetS in subject groups with differing BMIs. Therefore, we analysed the association of the WC and hip circumference individually with the risk of MetS using multivariable logistic regression analysis. The model also included age as a possible confounding variable; the results are shown in Table 4. These data clearly show that the WC is significantly (*p* < 0.0001) associated with an increased risk of MetS, whilst hip circumference is significantly (*p* = 0.019) associated with a reduced risk of MetS.

### 3.4. Use of WHR to Diagnose MetS

The data from Table 2 and Table 4 suggest that the WHR may be a suitable alternative to the WC to diagnose MetS in this population. To analyse this in more detail, the prevalence of MetS, diagnosed using the harmonised criteria but exchanging the WC ≥ 80 cm with the WHR > 0.84, was calculated and was shown to be 34.4% as compared to 49.6% when using the WC ≥ 80 cm (see Table 1). Subjects who were diagnosed with MetS using both of these criteria (*n* = 219) and those who had MetS diagnosed using only WC (*n* = 96) plus subjects without MetS were compared for anthropometric and cardiometabolic variables, and the results are shown in Table 5. There were just two subjects who were diagnosed with MetS using only the WHR criteria and due to this low sample size they were excluded from the analysis.

These data show that women diagnosed with MetS using only the WC criteria had a less severe form of MetS. Thus, these subjects had fasting glucose and triglyeride levels that were not significantly different from subjects without MetS, but both these variables were significantly lower (*p* < 0.0005 for both) when compared to those diagnosed with MetS using both the WC- and WHR-containing criteria. In addition, insulin resistance was also significantly lower (*p* < 0.005) in the former compared to the latter MetS group. All cardiometabolic variables were significantly higher (*p* < 0.0005 for all) in the subjects diagnosed with MetS using both the WC- and WHR-containing criteria when compared to the subjects without any form of MetS (see Table 5). In terms of the anthropometric variables, both subject groups with MetS had similar BMI levels, but those with only WC-diagnosed MetS had a significantly lower ABSI, WC, and WHR (*p* < 0.0005 for all) and a higher hip circumference (*p* < 0.005) when compared to subjects diagnosed with MetS using both the WC- and WHR-containing criteria.

## 4. Discussion

Using data from a representative sample of black South African women living in Soweto, Johannesburg, our analysis demonstrated that the WC cut-point for diagnosing MetS in this population increases with rising BMI, whereas the WHR and ABSI cut-points, defined using ROC curve analysis, did not change with the higher BMI. Furthermore, the AUC from the ROC curve and the sensitivity for the WHR and ABSI diagnoses of MetS were comparable to or better than those obtained when using either the WC cut-point or cut-points derived for other anthropometric variables, such as BMI or WHtR. The specificity of the MetS diagnosis using the WHR was higher than that of the majority of other anthropometric variables, whereas for ABSI it was lower. In addition, subjects diagnosed with MetS using both the WC- and the WHR-containing criteria were found to have a more severe cardiometabolic profile than those who were only diagnosed with MetS using the WC-containing criteria. These data suggest that the WHR and possibly the ABSI are suitable alternative anthropometric measures for the detection of MetS in this population.

The harmonised criteria for the detection of MetS recommends country-specific WC cut-points; however, as very little data is available, most countries in the SSA region follow the European guidelines for WC [13]. Our data and other studies show that the Europid-derived WC cut-point for the detection of MetS (80 cm) is not appropriate for African females and should be closer to 94 cm [4,5,11,14,15]. The main anthropometric driver of MetS and its cardiometabolic components is visceral fat, of which WC is a proxy measure [16,17], and studies have observed that the visceral fat depot is greater in European than African and African-American women [18,19,20]. This may be one of the reasons why WC cut-points derived from European populations are not appropriate in SSA. This contrasts with a meta-analysis using pooled SSA data, where the waist threshold for women was similar to that proposed by the harmonized guidelines [4]. However, this study observed that the cut-point varied widely across SSA with populations with high prevalence levels of obesity, such as South Africa, having higher WC cut-points for MetS diagnosis. This supports the results from the current study, which show that the WC cut-point for MetS rises with increasing BMI. These findings correspond with data from a study in Cape Town [14] and from studies conducted in populations living in Venezuela [21], Australia and Mauritius [16], and the USA [17], which all show that the WC cut-point for diagnosing MetS increases with BMI.

In our study, the AUC for the WHR from the ROC curve analysis and the sensitivities and specificities for the MetS diagnosis were similar to or better than those for the WC and the DXA-derived anthropometric measures. These findings are supported by a study from Korea, which demonstrated that WC, WHtR, and BMI were weaker predictors of metabolic diseases compared with the WHR [22], and the evidence supports the use of the WHR as a practical and easily accessible alternative for detecting diseases in both adolescents and adults [23,24]. The ROC curve analyses showed that trunk fat had similar performance to the WHR for the diagnosis of MetS in women with lower BMI, but performed slightly less well than the WHR in the high BMI group. In addition, a trunk fat measurement requires a DXA, which would render a MetS diagnosis near unattainable in under-resourced environments, such as those observed in sub-Saharan Africa. A further advantage to the use of the WHR for diagnosing MetS is that the cut-point does not change with increasing BMI. This is due to the protective metabolic effect of gluteofemoral fat [25], of which hip circumference is a proxy measure, which may counteract the negative metabolic effect of visceral fat as assessed via the WC. These opposing effects of waist and hip fat on cardiometabolic health are demonstrated in the logistic regression model for MetS (Table 4). Consequently, as BMI increases, so do WC and hip circumference, and the opposing effects of these depots on the metabolism mean that with increasing BMI, a higher WC is required to detect MetS due to the counter effect of the hip. In addition, our data demonstrate that subjects who are diagnosed with MetS only by the criteria that includes WC have less severe cardiometabolic disease compared to those that are diagnosed with MetS by both the WHR- and WC-containing criteria. This latter group of subjects has a greater WC but a lower hip circumference than the former group, which may explain their poorer cardiometabolic profile. It is possible that gluteofemoral fat may have greater effects in African females due to their higher levels of this fat depot [26], but it would be interesting to determine if the strength of the relationship between the WHR and the MetS risk varies across population groups.

A Body Shape Index is an allometric measure of body fat distribution that is independent of BMI [12]. It has been shown to complement BMI and to perform better than other measures of abdominal obesity in mortality risk stratification in a large longitudinal study of all-cause mortality [27]. Therefore, the ABSI was analysed in the current study and, similarly to the WHR, it provided cut-points for the diagnosis of MetS that were not altered by variations in BMI. Furthermore, the ABSI cut-point generated for the diagnosis of MetS had a much higher sensitivity than any of the other anthropometric variables, but it had a lower specificity. These data suggest that the ABSI and WHR may both be possible alternatives to WC as one of the criteria for the diagnosis of MetS in SSA women. Studies from SSA are, therefore, required to compare the ability of the WC, WHR, and ABSI to predict future cardiometabolic diseases and also to determine whether MetS diagnosed using each of these anthropometric variables differs in its ability to predict CVD events.

The main limitation of this study was that it was cross-sectional, which means that causative associations can only be inferred. In addition, the study used only women, but this decision was made because obesity is much more prevalent in women than in men in SSA, and the female population used were known to have a high prevalence of the metabolic syndrome [8]. The large sample size, the comparison of simple and more advanced obesity indices, and the use of a well-characterized but under-investigated population with a high prevalence of MetS added strength to this study.

## 5. Conclusions

Our findings and those of other studies confirm that the current WC cut-point recommended for the diagnosis of MetS in SSA women is not appropriate and that it increases with rising BMI. Urgent revisions of the current guidelines for MetS are, therefore, needed to ensure that accurate epidemiological data are used for public health initiatives addressing obesity and associated cardiometabolic diseases in the SSA region. Our study provides a novel scientific evidence for the WHR or ABSI as alternate anthropometric measures for the detection of MetS in SSA women.

## Figures and Tables

**Table 1:** Subject characteristics stratified by the median BMI and for the whole cohort.

Variables	BMI < 32.8 kg/m^2^ (N = 354)	BMI ≥ 32.8 kg/m^2^ (*n* = 348)	Combined (*n* = 702)
**Anthropometric and cardiometabolic variables**			
ABSI	0.126 ± 0.01	0.129 ± 0.01 ***	0.127 ± 0.01
BMI (kg/m^2^)	27.7 ± 3.80	38.9 ± 5.44 ***	33.4 ± 7.32
WC (cm)	89.5 ± 10.2	109 ± 11.5 ***	99.1 ± 14.5
WHR	0.83 ± 0.08	0.85 ± 0.08 **	0.84 ± 0.07
WHtR	0.57 ± 0.07	0.68 ± 0.06 ***	0.62 ± 0.08
Arm + leg fat (kg)	14.3 ± 3.76	21.7 ± 4.58 ***	17.9 ± 5.57
Trunk fat (kg)	10.9 ± 3.41	18.2 ± 3.69 ***	14.5 ± 5.09
Sub-total body fat (kg)	25.2 ± 6.46	39.8 ± 7.18 ***	32.4 ± 10.0
Systolic BP (mmHg)	129 (117, 145)	134 (123, 147) **	131 (119, 146)
Diastolic BP (mmHg)	84.5 (77.0, 92.0)	89.5 (83.0, 99.5) ***	87.0 (79.0, 96.0)
Fasting glucose (mmol/L)	4.70 (4.40, 5.10)	4.90 (4.50, 5.30)	4.80 (4.50, 5.20)
HDL (mmol/L)	1.30 (1.00, 1.50)	1.10 (0.90, 1.30) ***	1.20 (1.00, 1.40)
Triglycerides (mmol/L)	1.00 (0.80, 1.40)	1.20 (0.80, 1.50)	1.10 (0.80, 1.50)
**Prevalence of MetS and component disorders**			
WC ≥ 80 cm (%)	81.2 (77.0, 85.3)	99.7 (99.2, 100) ***	89.7 (87.4, 91.9)
Systolic BP ≥ 130 and/or diastolic BP ≥ 85 mmHg (%)	55.7 (50.4, 60.9)	73.8 (69.1, 78.4) ***	65.5 (61.4, 68.6)
Fasting glucose ≥ 5.6 mmol/L (%)	13.7 (9.91, 17.4)	18.9 (14.7, 23.2)	16.5 (13.6, 19.4)
HDL < 1.3 mmol/L (%)	50.0 (44.6, 55.4)	70.1 (65.1, 75.2) ***	59.7 (56.0, 63.5)
Triglycerides ≥ 1.7 mmol/L (%)	13.8 (10.1, 17.6)	16.9 (12.9, 21.0)	15.4 (12.6, 18.1)
MetS (with WC) (%)	36.4 (31.2, 41.7)	63.1 (57.8, 68.4) ***	49.6 (45.7, 53.5)
MetS (excluding WC) (%)	9.00 (5.88, 12.1)	19.1 (14.8, 23.4) ***	14.0 (11.3, 16.7)

Data expressed as a mean ± SD, median (interquartile range), or % (95% Cis); ** *p* < 0.005; *** *p* < 0.0005 versus subjects below the median for body mass index (BMI); ABSI, A Body Shape Index; WC, waist circumference; WHR, waist-to-hip ratio; WHtR, waist-to-height ratio; BP, blood pressure; HDL, high density lipoprotein cholesterol; MetS, metabolic syndrome.

**Table 2:** Optimal cut-points for detecting MetS (without WC) using simple anthropometric indices in subjects with BMI below or above median value.

Anthropometric Variables	Cut-Point	AUC	Sensitivity	Specificity
**Women with BMI below median**				
ABSI	0.127	0.74 (0.66, 0.82) *	0.86 (0.82, 0.90)	0.38 (0.34, 0.42)
BMI (kg/m^2^)	29.3	0.61 (0.51, 0.71) *	0.66 (0.61, 0.71)	0.44 (0.39, 0.49)
WC (cm)	93.5	0.74 (0.65, 0.82) *	0.67 (0.60, 0.75)	0.52 (0.45, 0.59)
WHR	0.85	0.72 (0.63, 0.82) *	0.70 (0.67, 0.73)	0.52 (0.49, 0.55)
WHtR	0.58	0.71 (0.61, 0.81) *	0.69 (0.66, 0.72)	0.52 (0.49, 0.55)
**Women with BMI above median**				
ABSI	0.129	0.59 (0.52, 0.68) *	0.66 (0.62, 0.70)	0.45 (0.41, 0.49)
BMI (kg/m^2^)	35.9	0.54 (0.45, 0.62) *	0.60 (0.55, 0.64)	0.43 (0.39, 0.48)
WC (cm)	107	0.58 (0.50, 0.66) *	0.53 (0.46, 0.61)	0.53 (0.46, 0.60)
WHR	0.84	0.62 (0.54, 0.69) *	0.59 (0.56, 0.63)	0.52 (0.49, 0.55)
WHtR	0.66	0.56 (0.47, 0.64) *	0.55 (0.51, 0.58)	0.51 (0.48, 0.55)

Data in parentheses are 95% confidence intervals; * *p* < 0.05 for area-under-the-curve versus (AUC) = 0.5; ABSI, A Body Shape Index; BMI, body mass index; WHR, waist-to-hip ratio; WHtR, waist-to-height ratio; WC, waist circumference.

**Table 3:** Optimal cut-points for detecting MetS (without WC) using DXA-derived anthropometric indices in subjects with BMI below or above median value.

Anthropometric Variables	Cut-Point	AUC	Sensitivity	Specificity
**Women with BMI below median**				
Subtotal fat (kg)	25.9	0.59 (0.49, 0.68) *	0.58 (0.54, 0.61)	0.51 (0.48, 0.54)
Arm and leg fat (kg)	15.1	0.49 (0.39, 0.58)	0.49 (0.45, 0.52)	0.50 (0.47, 0.53)
Trunk fat (kg)	12.1	0.70 (0.60–0.79) *	0.68 (0.64, 0.71)	0.52 (0.49, 0.55)
**Women with BMI above median**				
Subtotal fat (kg)	39.5	0.57 (0.49, 0.66) *	0.56 (0.53, 0.59)	0.51 (0.48, 0.54)
Arm and leg fat (kg)	11.0	0.37 (0.28, 0.46)	0.40 (0.37, 0.43)	0.47 (0.44, 0.51)
Trunk fat (kg)	13.8	0.52 (0.44, 0.60) *	0.52 (0.48, 0.55)	0.50 (0.47, 0.54)

Data in parentheses are 95% confidence intervals; * *p* < 0.05 for hypothesis test of whether AUC > 0.5; SAT, subcutaneous adipose tissue; VAT, visceral adipose tissue.

**Table 4:** A multivariable logistic regression model illustrating the relationship between waist and hip circumferences and metabolic syndrome (without waist).

Dependent Variable	Independent Variables	Odds Ratio (95% CIs)	*p*-Value
Metabolic syndrome	Age	1.07 (1.02, 1.12)	0.003
	Waist circumference	1.07 (1.04, 1.10)	<0.0001
	Hip circumference	0.97 (0.94, 0.99)	0.019

**Table 5:** Anthropometric and cardiometabolic data of subjects without metabolic syndrome, with metabolic syndrome diagnosed by waist only, and with metabolic syndrome diagnosed by both waist and waist-to-hip ratio.

Variables	No MetS (*n* = 322)	MetS by WC (*n* = 96)	MetS by WC & WHR (*n* = 219)
Age (years)	48.5 ± 5.14	49.8 ± 5.13	50.2 ± 5.40 **
ABSI	0.125 ± 0.01	0.123 ± 0.01	0.133 ± 0.01 ***^,†††^
BMI (kg/m^2^)	31.0 ± 6.93	36.0 ± 6.27 ***	35.2 ± 6.72 ***
WC (cm)	93.6 ± 14.3	98.6 ± 9.09 ***	106 ± 11.9 ***^,†††^
Hip (cm)	114 ± 14.4	125 ± 13.8 ***	120 ± 12.8 ***^,††^
WHR	0.82 ± 0.07	0.79 ± 0.04 ***	0.89 ± 0.05 ***^,†††^
Systolic BP (mmHg)	121 (113, 136)	136 (130, 145) ***	140 (128, 154) ***
Diastolic BP (mmHg)	81.0 (75.0, 91.0)	90.7 (85.7, 99.0) ***	91.5 (86.5, 100) ***
Fasting glucose (mmol/L)	4.60 (4.30, 4.90)	4.85 (4.50, 5.20)	5.10 (4.70, 5.80) ***^,^^†††^
HDL (mmol/L)	1.40 (1.10, 1.60)	1.10 (0.90, 1.20) ***	1.00 (0.90, 1.10) ***
Triglycerides (mmol/L)	1.00 (0.70, 1.20)	1.10 (0.80, 1.35)	1.35 (1.00, 1.80) ***^,†††^
HOMA-IR	1.76 (1.21, 2.69)	2.31 (1.47, 3.17) **	2.88 (1.97–4.82) ***^,^^††^

Data expressed as a mean ± SD or median (interquartile range); ** *p* < 0.005; *** *p* < 0.0005 versus subjects without metabolic syndrome (MetS); ^††^ *p* < 0.005; ^†††^ *p* < 0.0005 versus subjects with MetS by WC (waist circumference); ABSI, A Body Shape Index; BMI, body mass index; BP, blood pressure; HDL, high density lipoprotein cholesterol; HOMA-IR, homeostasis model assessment-insulin resistance; WHR, waist-to-hip ratio.

## Data Availability

Anonymized study data are available upon motivated and reasonable request to the corresponding author.
